# Brain fMRI during orientation selective epidural spinal cord stimulation

**DOI:** 10.1038/s41598-021-84873-8

**Published:** 2021-03-09

**Authors:** Antonietta Canna, Lauri J. Lehto, Lin Wu, Sheng Sang, Hanne Laakso, Jun Ma, Pavel Filip, Yuan Zhang, Olli Gröhn, Fabrizio Esposito, Clark C. Chen, Igor Lavrov, Shalom Michaeli, Silvia Mangia

**Affiliations:** 1 Center for Magnetic Resonance Research (CMRR), Department of Radiology, University of Minnesota, 2021 6th St. SE, Minneapolis, MN 55455 USA; 2 Department of Advanced Medical and Surgical Sciences, University of Campania “Luigi Vanvitelli”, Naples, Italy; 3 A. I. Virtanen Institute for Molecular Sciences, University of Eastern Finland, Kuopio, Finland; 4 Department of Neurosurgery, University of Minnesota, Minneapolis, MN USA; 5 Department of Neurology, Charles University and General University Hospital in Prague, Prague, Czech Republic; 6 Division of Biostatistics, School of Public Health, University of Minnesota, Minneapolis, MN USA; 7 Department of Physiology and Biomedical Engineering, Mayo Clinic, Rochester, MN USA; 8 Department of Neurology, Mayo Clinic, Rochester, MN USA

**Keywords:** Preclinical research, Biomedical engineering, Magnetic resonance imaging, Neuroscience, Neural circuits

## Abstract

Epidural spinal cord stimulation (ESCS) is widely used for chronic pain treatment, and is also a promising tool for restoring motor function after spinal cord injury. Despite significant positive impact of ESCS, currently available protocols provide limited specificity and efficiency partially due to the limited number of contacts of the leads and to the limited flexibility to vary the spatial distribution of the stimulation field in respect to the spinal cord. Recently, we introduced Orientation Selective (OS) stimulation strategies for deep brain stimulation, and demonstrated their selectivity in rats using functional MRI (fMRI). The method achieves orientation selectivity by controlling the main direction of the electric field gradients using individually driven channels. Here, we introduced a similar OS approach for ESCS, and demonstrated orientation dependent brain activations as detected by brain fMRI. The fMRI activation patterns during spinal cord stimulation demonstrated the complexity of brain networks stimulated by OS-ESCS paradigms, involving brain areas responsible for the transmission of the motor and sensory information. The OS approach may allow targeting ESCS to spinal fibers of different orientations, ultimately making stimulation less dependent on the precision of the electrode implantation.

## Introduction

Conventional epidural spinal cord stimulation (ESCS) is commonly used for several diseases mostly associated with chronic pain conditions, complex regional pain syndrome, neuropathic and ischemic pain and painful diabetic peripheral neuropathy^1–3^. ESCS was introduced after the introduction of the gate control hypothesis^4^ according to which stimulating the low-threshold myelinated fibers in the dorsal columns of the spinal cord with electrical impulses at specific frequencies might lead to pain reduction^5^. In clinical practice, conventional low frequency ESCS induces paresthesia in a distribution overlapping with the painful area, thus reducing the perception of pain^3,6^. The success of traditional ESCS is largely based on the ability of the clinician to provide an adequate coverage over the patients’ distribution of pain and furthermore the willingness of the patient to tolerate the induced paresthesia^3^. Despite the significant positive impact of ESCS, current protocols are generally hampered by the limited number of contacts in the available leads and by their spatial distribution along the spinal cord^1^. Hence, these challenges can lead to the failure of ESCS therapy for patients reporting distributions of pain in regions that are difficult to cover with paresthesia^3^. In addition to pain treatment, ESCS is a promising tool for restoring motor function after spinal cord injury (SCI) in patients. For example, with different contact combinations, Gill and colleagues^7^ were able to restore independence of motor function after complete loss of lower sensorimotor functions after SCI. In another study, Wagner and colleagues re-established adaptive control of paralyzed muscles during walking by modulating the stimulation in both space and time^8^.

Areas of growing research involve the optimization and advancement of ESCS strategies beyond the conventional paradigms that use low frequency, high amplitude, and 300–600 μs pulse widths. Recently introduced monophasic burst paradigms^9^ have been shown to result in clinically relevant pain reduction, without eliciting paresthesia, especially for the treatment of pain with lumbosacral relay component^10^. However, while some studies reported a clear superiority of burst ESCS in respect to conventional ESCS^9,11,12^, another study failed to find significant differences among the two approaches^13^. Stimulations at high frequency (~ 10 kHz) employing pulses with 30 μs duration and 1–5 mA amplitudes also hold promise for ESCS, as they do not require intraoperative mapping contrary to the low-frequency strategies^14^. However, they still need rechargeable batteries, an eventual inconvenience and discomfort for the patients. A possible alternative for reducing energy requirements and thus enabling non-rechargeable batteries is to stimulate the dorsal roots directly, but more demanding implantation procedures are required in such case for placing the electrode leads on the dorsal roots themselves and thus steering the electrical field in space along their directions^15^.

Rather than activating the electrode leads physically placed along the axonal pathway of interest, one can target specific axonal pathways passing by or in close vicinity to the implantation site by reorienting the electrical field gradient with independently controllable channels, an approach that we termed orientation selective (OS) stimulation^16,17^. In order to achieve full reorientation flexibility of the electrical field gradients on a plane, a minimum of only 3 independently controllable channels distributed along a triangle is needed, while 3D reorientation capabilities can be achieved with a minimum of 4-channel electrodes where one lead is placed off-plane from the other three. The OS strategy was recently successfully used in deep brain stimulation (OS-DBS) of rodents for the stimulation of the well-established fiber pathway encompassing the corpus callosum^17^ and for more complex circuitries encompassing the infralimbic cortex^16^ or the subthalamic nucleus^18^. Furthermore, based on patient specific models, OS-DBS was demonstrated to be effective for enhancing activation of specific networks of interest for Parkinson’s disease^19^ also when using commercially available multichannel electrodes with independently driven contacts. A similar conceptual framework of OS stimulation is applicable to ESCS. Most epidural electrodes in clinical practice employ multiple channels that are distributed along a paddle and are driven by a single current source. As such, they allow steering flexibility (i.e., directing the electrical field in space to the target of interest), but only limited reorientation flexibility of the electrical field spatial gradients. In fact, each channel can be switched only between cathode and anode, and the resulting electrical field direction is limited to those imposed by the geometrical distribution of the contacts. On the other hand, employing multiple independent current sources to deliver unequal amounts of current through the leads in principle allows reorienting the electrical field gradient with much higher angular resolution. The use of multiple current sources is being increasingly exploited in the SCS field^20–22^, and advanced electrodes with multiple independently controllable leads such as the Illumina3D system from Boston Scientific have recently became available for ESCS, thus opening unprecedented opportunities for OS-ESCS in clinical applications.

The purpose of the current study was to gain initial insights into the effects of OS-ESCS in rodents. To achieve this goal, we first carried out monopolar ESCS experiments to identify an optimal stimulation frequency; then we used 3-channel electrodes with independently driven channels and variable amplitudes for each channel to control the primary direction of the electric field gradient on a plane. As stimulation sites, we chose two spinal segments that are critical for the recovery and integration of sensorimotor functions as they elicit optimal locomotor activity^23^ and impact stepping performance^24^, namely the sacral (S1) and rostral lumbar (L2) spinal segments. In particular, the present investigation was designed to test the impact of the stimulation angle on the integrative central nervous system (CNS) response as measured by brain activation patterns with functional MRI (fMRI). Finite element method simulations were also conducted to illustrate the behavior of the electric field on spinal cord when using OS-ESCS. We hypothesized that significant modulation of activation patterns would be observed at both single subject and group levels as a function of the stimulation angle due to the activation of different bundles of axons. In particular, we expected that OS-ESCS along the direction of the spinal cord would induce maximal activation of the brain regions of interest that are responsible for the transmission of the motor and sensory information, including the thalamus, the motor cortex and the primary somatosensory cortex, via direct or indirect connection to the spinal cord itself. Finally, we did not expect significant group differences when stimulating S1 vs L2 spinal segments given the relatively small sample size used in this pilot investigation.

## Results

**Figure 1:**
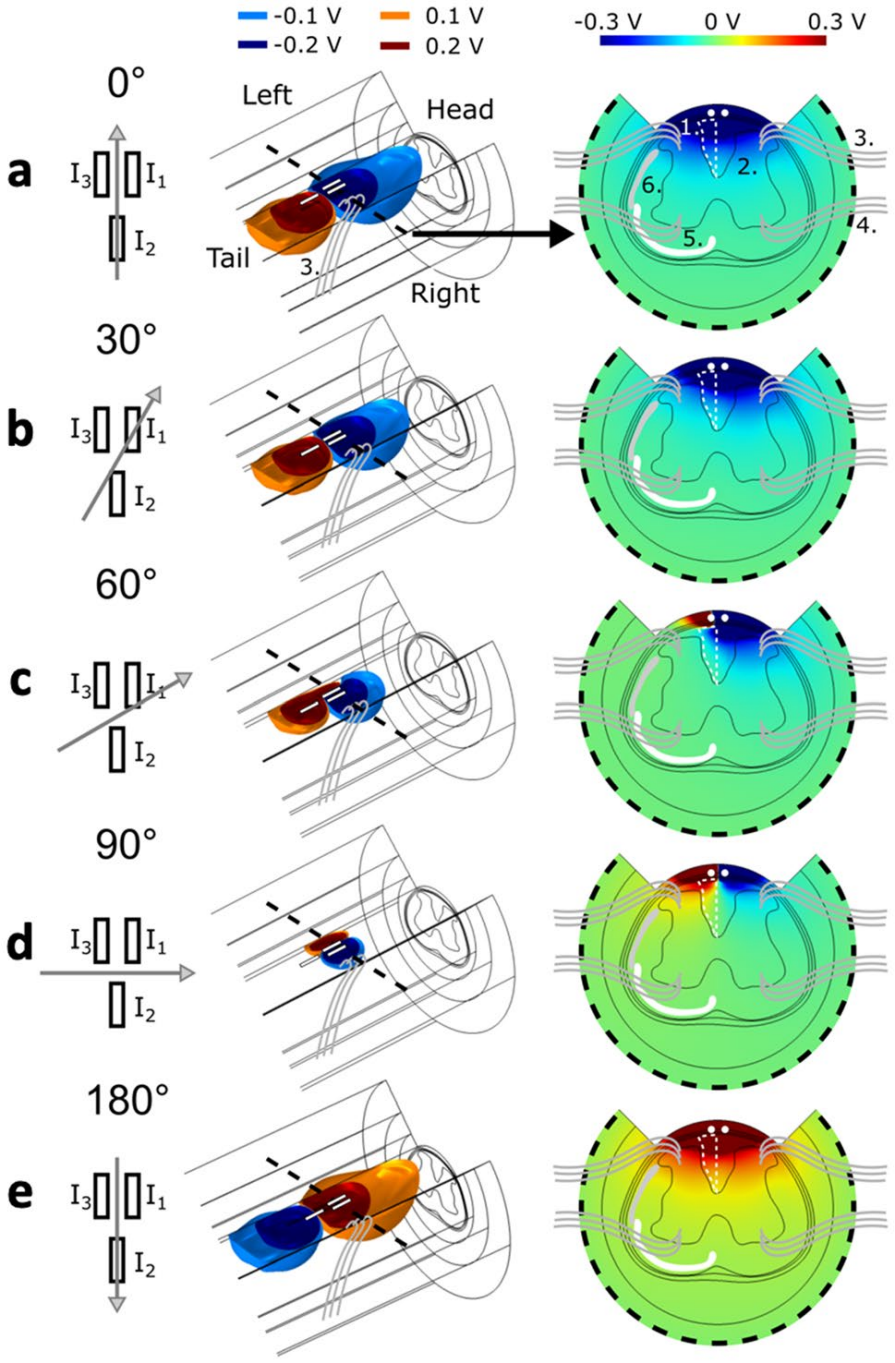
OS-ESCS in the rat spinal cord as seen by using finite element methods. The model was constructed based on Watson atlas using SolidWorks 2018 (Dassault Systèmes, Waltham, MA), after which the model was transferred to COMSOL 5.4 (COMSOL, Stockholm, Sweden) for simulations of electrical field potentials. The model included a section of an anatomically correct rat spinal cord surrounded by cylindrical layers of CSF, dura, epidural fat, muscle and bone. (**a**–**e**) The behavior of the electric potential field using different OS-ESCS stimulation angles is shown using electrode schematics representing the stimulation direction, 3D isosurfaces and 2D surface plots across the two rostral electrode contacts. I_1,2,3_ in the first column correspond to the currents described by Eq. (1). The location of the main structures relevant to ESCS are illustrated in (**a**): 1. dorsal columns, 2. dorsal horn, 3. dorsal roots, 4. ventral roots, 5. Spinothalamic tract and 6. spinocerebellar tract.

Results of the finite element method simulation are shown in Fig. 1 for different stimulation angles of the OS-ESCS approach. Various electric potential field distributions and degrees of field penetration can be appreciated depending on the direction of the electrical field, implying that the activation of the main spinal cord structures can be modulated by varying the stimulation angle.

The high-resolution CT scans performed for documenting the actual location of the electrode in some animals confirmed that the electrodes were implanted at the desired vertebra level, although small lateral variations in the order of few millimeters were observed (Supplementary Figure 1). For the fMRI scans, none of the analyzed rats (and related functional runs) showed a mean framewise displacement value above threshold for exclusion. Current amplitudes were between 0.15–0.6 mA for monopolar ESCS, between 0.6–1 mA for OS-ESCS with stainless steel electrodes placed on the S1 spinal segment, and between 1.5–3.25 mA for OS-ESCS with tungsten electrodes placed on the L2 spinal segment.

**Figure 2:**
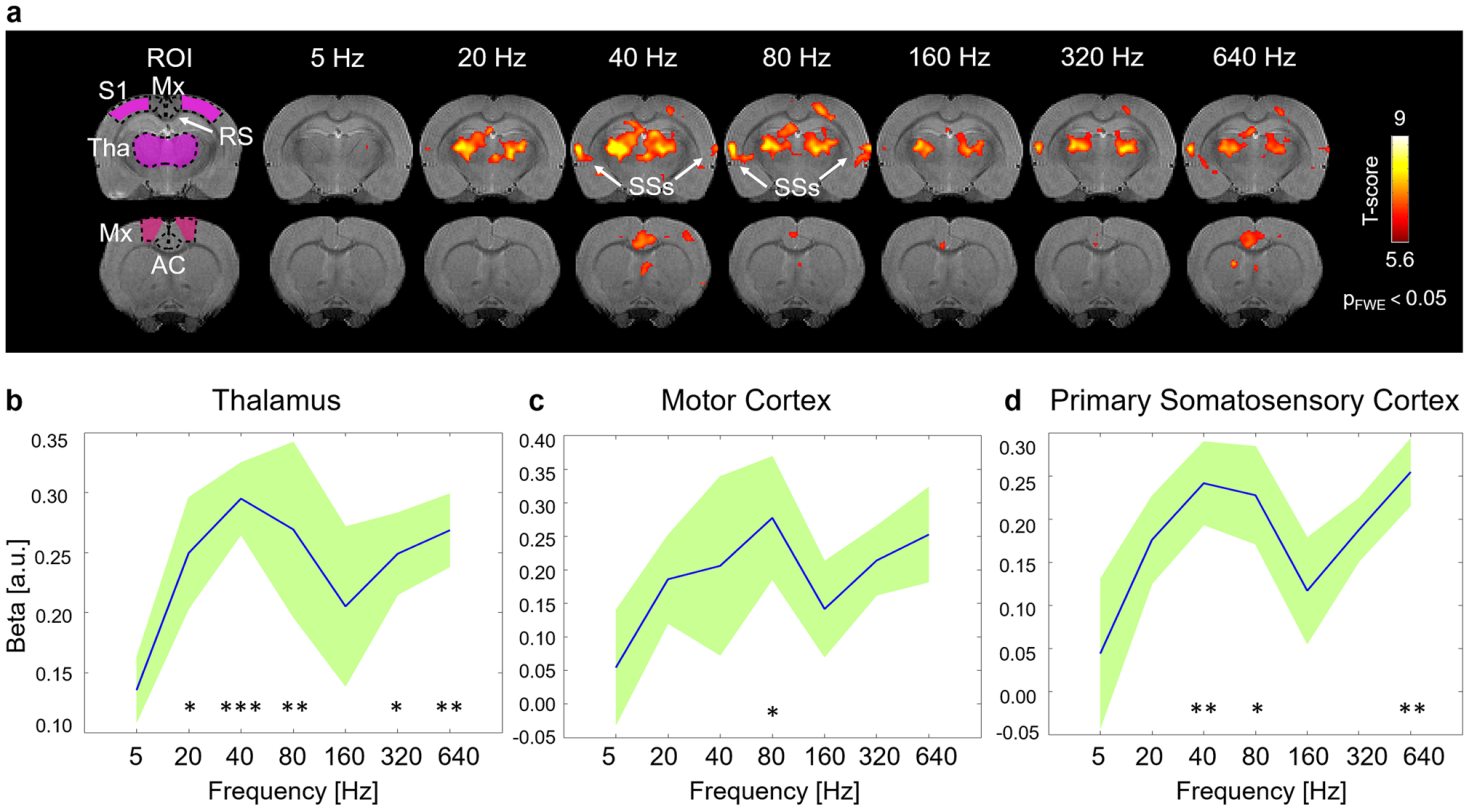
Group level responses to single contact/wire monopolar ESCS at different stimulation frequencies. (**a**) Group level maps of the main effects (t-maps) in two different brain slices. Maps were obtained by the one-way within subject ANOVA model (p < 0.05 family wise corrected (FWE) n = 6). ROI-averaged beta values in the thalamus (**b**), the motor cortex (**c**) and the primary somatosensory cortex (**d**) in response to different stimulation frequencies. Blue line indicates mean while green shading indicates the standard deviation. Holm’s corrected *p < 0.05, **p < 0.01, ***p < 0.001 (linear mixed model comparisons vs 5 Hz). *S1* primary somatosensory cortex, *Mx* motor cortex (including primary and secondary motor cortices), *RS* retrosplenial, *Tha* thalamus, *AC* anterior cingulate cortex, *SSs* supplementary somatosensory cortex. Brain images are displayed in neurological convention (left side of the image corresponds to the left side of the brain).

In Fig. 2a, group level activation maps during monopolar stimulation of the SC at different frequencies are shown. Activations in the thalamic area were detected consistently with all stimulation frequencies except for that at 5 Hz, which was used as a reference for comparisons. More spread pattern of activations were observed with monopolar stimulation at 40 Hz and 80 Hz, including not only the anterior cingulate cortex and the thalamus but also the motor cortex, the primary somatosensory cortex and the supplementary somatosensory cortex. Linear mixed model analysis revealed significant differences after Holms’ correction between activations at 5 Hz vs 20 Hz (p = 0.02), 40 Hz (p = 0.0009), 80 Hz (p = 0.007), 320 Hz (p = 0.02) and 640 Hz (p = 0.007) in the thalamus (Fig. 2b), between 5 Hz vs 80 Hz (p = 0.026) in the motor cortex (Fig. 2c) and between 5 Hz vs 40 Hz (p = 0.006), 80 Hz (p = 0.01) and 640 Hz (p = 0.004) in the somatosensory cortex (Fig. 2d). As mentioned in the method section, a frequency of 40 Hz was ultimately set for performing the OS-ESCS experiments.

**Figure 3:**
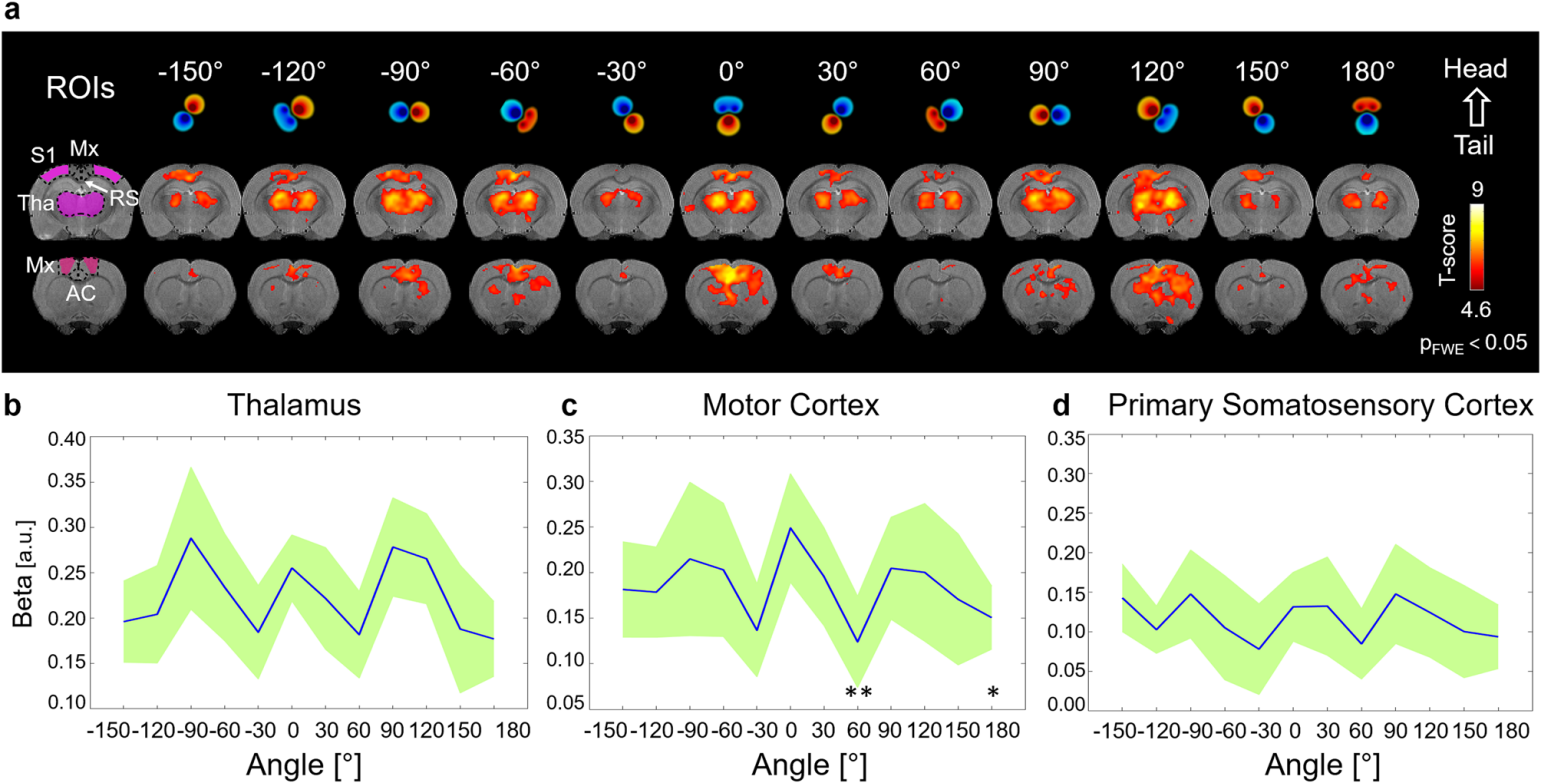
Group level responses to OS-ESCS with 3-channel electrodes. (**a**) Group level maps of the main effects (t-maps) in two different brain slices. Maps were obtained by the one-way within subject ANOVA model (p < 0.05 FWE corrected, n = 11). ROI-averaged beta values in the thalamus (**b**), in the motor cortex (**c**) and in the primary somatosensory cortex (**d**) in response to different stimulation angles. Blue line indicates mean while green shading indicates the standard deviation. Holm’s-corrected *p < 0.05, **p < 0.01 (linear mixed model comparisons vs 0°). *S1* primary somatosensory cortex, *Mx* motor cortex (including primary and secondary motor cortices), *RS* retrosplenial, *Tha* thalamus, *AC* anterior cingulate cortex. Brain images are displayed in neurological convention (left side of the image corresponds to the left side of the brain).

The voxel-wise group analysis of the data obtained from the whole group of OS-ESCS studies (n = 11) with either S1 and L2 spinal segment stimulations demonstrated significant activations in the thalamus, in the motor cortex, in the primary somatosensory cortex, as well as in other areas including retrosplenial, and anterior cingulate cortex (Fig. 3a). Highly similar main effects were observed also in the group analyses without considering the stimulation currents and/or the experimental setups (namely, setup 1: S1 spinal segment stimulation with stainless steel electrodes, and setup 2: L2 spinal segment stimulation with tungsten electrodes) as covariate of no interest (Supplementary Figure 2). Modulation of activation patterns were clearly visible as a function of the stimulation angle both in single subjects (Supplementary Figures 2 and 3) and at the group level (Fig. 3a). fMRI signal change plots in the thalamus, sensory cortex and motor cortex show mean activations between ~ 1% and ~ 2% with respect to the baseline as shown in the Supplementary Figure 4. Angular modulations were confirmed in the ROI analyses of anatomically defined regions of the thalamus, motor cortex and primary somatosensory cortex (Fig. 3b–d), while the linear mixed model revealed significant group difference between the activations of the motor cortex at 0° vs 60° (Holm’s corrected p = 0.004) and vs 180° (Holm’s corrected p = 0.049).

**Figure 4:**
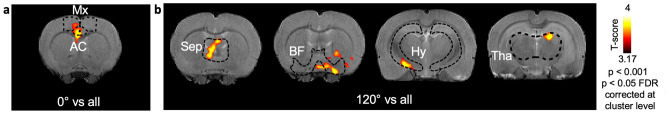
Contrast analysis of OS-ESCS. Contrast t-maps showing the significant clusters obtained by the analyses 0° vs all (**a**) and 120° vs all (**b**). Only contrasts providing significant results are shown. The t-maps have been produced with a statistical threshold of p < 0.001 at voxel level (n = 11). The cluster survived the statistical threshold of p < 0.05 FDR corrected. *Mx* motor cortex (including primary and secondary motor cortices), *AC* anterior cingulate, *BF* basal forebrain regions, *Hy* hippocampus, *Sep* septal regions, *Tha* thalamus.

The one-vs-all contrast analyses produced clusters of significant differences between the activation at 0° vs all the other angles (Fig. 4a), and between 120° vs all other angles (Fig. 4b). The cluster obtained from the contrast 0° vs all was in the region including the anterior cingulate cortex and the motor cortex (T = 4.62, p = 0.008 false discovery rate (FDR) corrected at cluster-level, cluster size 160 voxels). The clusters obtained from the contrast analysis 120° vs all were located in the basal forebrain regions (T = 4.66, p = 0.00003 FDR corrected at cluster level, cluster size 445 voxels), in the hippocampal formation (T = 4.48, p = 0.004 FDR corrected at cluster level, cluster size 203 voxels), in a region encompassing the basal forebrain region and the septal region (T = 4.29, p = 0.018 FDR corrected at cluster level, cluster size 140 voxels), and in a region localized in the thalamus (T = 4.43, p = 0.037 FDR corrected at cluster level, cluster size 108 voxels).

In order to denoise the data from potential fluctuations induced by anesthesia, we also repeated the main analyses after extracting independent components. The main effects obtained by the group Indipendent Component Analysis (ICA) (Supplementary Figure 6) show consistent activation in the thalamus, motor and somatosensory cortex, although they occurred at a lower statistical threshold and with smaller cluster sizes due to the ICA-related dimensionality reduction. This observation excluded significant biases induced by urethane.

Finally, we explored whether different activation patterns could be detected when stimulating S1 vs L2 spinal segments in our sample. From the flexible factorial analysis, we did not obtain any clusters of significant group effect or interactions (group by angle interactions) effect at a statistical threshold of p < 0.05 family wise error (FWE) corrected and at a lower statistical threshold p < 0.001 uncorrected at voxel level and p < 0.05 FDR corrected at cluster level. The ANOVA analysis at regional level did not reveal any significant group and interaction (group by angle) effects at p < 0.05 FDR corrected.

## Discussion

In this pilot investigation we introduced a novel approach for ESCS, namely OS-ESCS, which allows changing the primary direction of the electric field by independently varying the current amplitudes of each channel of a 3-channel electrode. To demonstrate the ability of OS-ESCS to modulate spinal-brain connectomes within the CNS, we monitored the brain activations during OS-ESCS using whole-brain fMRI in rats during stimulation of two spinal segments critical for locomotor activity, namely the S1 and L2 spinal segments. Two types of electrodes with different mechanical and electrical properties, i.e., stainless steel and tungsten, were used to stimulate the S1 and L2 spinal segments, respectively. Importantly, whereas tungsten wires necessitated higher current amplitudes to achieve thalamic activations as compared to stainless steel wires, the two electrode types and locations, as well as the stimulation currents per se, did not affect the main group effects of OS-ESCS at the various stimulation angles.

Clusters of activation in various brain regions were clearly observed during OS-ESCS, including the thalamus and motor cortex, as well as the primary somatosensory cortex, retrosplenial, anterior cingulate cortex. Despite unavoidable lateral variations in electrode locations (Supplementary Figure 1), the loci of significant activations were consistent among single subjects, irrespective of S1 vs L2 spinal segment electrode implantations (Supplementary Figures 3 and 4), whereas the spread and angle dependence of the activation patterns was variable among subjects. The observed pattern of activation demonstrates a consistent involvement of brain areas responsible for direct or indirect transmission of sensory and motor information from the spinal cord to the brain network via the thalamus and related cortices. Strong orientation selectivity occurred in the cingulate and motor cortices, brain areas highly connected or belonging to the motor network^25^, as revealed by voxel-wise contrast analysis between 0° and all other angles (0° vs all contrast). Strong orientation selectivity was also observed when analyzing the contrast between 120° vs all other angles in areas within the basal forebrain and septal regions, the thalamus and the hippocampus. These two directions (0° and 120°) have an important geometrical valence since 0° is the direction parallel to the spinal cord whereas 120° is the closest direction to the dorsal roots, the first hub of the sensory pathway.

ESCS and brain fMRI studies have been done in humans^5,26–29^. In such earlier studies, negative activations of the thalamus, cingulate cortex and insula were seen in failed back surgery patients^28^, but also negative and positive activations have been reported in the somatosensory cortex while having negative activation in the primary motor cortex^5^. ESCS and brain fMRI studies have been conducted also in rats^30,31^. Upon stimulation of the dorsal root ganglion of a rat, nearly no brain fMRI response was observed with a therapeutic level stimulus, although at higher stimulus positive activation of the thalamus and negative activation of the caudate and putamen were seen, corresponding to noxious stimuli^31^. In chronic neuropathic rats, activation of cortical and subcortical brain areas like the thalamus, the anterior cingulate cortex as well as the motor and sensorimotor cortices was observed during conventional and burst SCS^30^. Such areas resemble the outcomes presented in the current study, therefore in the sense of elicited activation patterns, our results are in line with existing literature.

Using OS-ESCS, one might expect a symmetric response using stimulation angles differing by 180°^17^. Despite the considerable variability observed across subjects (Supplementary Figures 3 and 4), the ROI group analyses (Fig. 3b) reveal a stimulation angle dependence that is mostly consistent with such symmetry. Yet, for instance thalamic and motor cortex responses appear to be lower at 180° as compared to 0°. Such finding can be explained by noting that exact symmetry cannot occur with most stimulation angles when using 3 channels along a triangle configuration (Fig. 1). At OS-ESCS angle of 0° the cathode is divided between two rostral contacts while at 180° it is concentrated on the single caudal contact. Hence at stimulation angle of 0° the cathodic field extends wider mediolaterally towards the dorsal root entry zones while not penetrating as deep into the dorsal columns in comparison to the stimulation angle of 180°, where the cathodic field extends further into the dorsal columns and less so towards the dorsal roots. In addition, at 180°, the two rostral contacts may act as partial anodic guard further decreasing activity initiated in the dorsal roots. Near exact symmetry of the electric field for stimulation angle pairs with a 180° difference can only be achieved with − 90°/90° as only then the cathode and anode are placed on single contacts. Such prediction was indeed reflected in our results where − 90°/90° gave similar level of responses especially in the thalamus (Fig. 3). Further sources for electric field asymmetry between different stimulation angles stem from the design of the lead itself. Namely, equilateral triangle arrangement of the contacts and the fact that the contacts themselves are long compared to their width. Thus, firstly, the distance between the anode and cathode are stimulation angle dependent so that at − 90° and 90° the distance is minimal, while at − 30°/150° and 30°/− 150° the distance is maximal and similar to 0° and 180°. Secondly, at 0° and 180° the cathodic and anodic fields emanate from the contacts with their short axis facing each other while at − 90° and 90° the long axes of the contacts face each other. These two considerations together mean that at 0° and 180° the dipole field is the most disperse and at − 90° and 90° the field is the most concentrated (Fig. 1). Hence, the electric field at 0° and 180° penetrates the tissue deeper, but on the other hand − 90° and 90° may induce stronger 2nd derivatives of the potential field^32^ in the anterior dorsal columns or at the entry zones of the dorsal roots. This could mean that at − 90° and 90°, even though the orientation of the field might not be optimal, action potentials are still induced at a similar level, which could partly explain similar group level responses when comparing stimulation angles 0° and − 90°/90°. The electric field behavior of other stimulation angles is then between the two extrema of 0° and 180° and − 90° and 90°.

An important source of variation in the obtained fMRI response could be also due to small variations in electrode alignment relative to the spinal cord, as observed in the 4 CT scans of rats implanted in the L2 spinal segment (Supplementary Figure 1). Importantly, even minor variations in electrodes placement could translate into substantial relative positional differences to the dorsal structures^33^. Especially the differences between stimulation angles with opposite signs are likely related to lateral shifts of the electrodes: if the electrode is not on the midline the stimulating field does not have lateral symmetry and different structures are stimulated with opposite sign stimulation angles. Moreover, significant differences in stimulation current was seen between OS-ESCS groups using stainless steel or tungsten electrodes. This may be related to tungsten being stiffer than stainless steel: stiffer wire is more susceptible to bowing away from the dura if the dura is not entirely flat and/or pressure is applied to the wire from touching bone and other tissue along its length. If the wire does lift above the dura (though still in saline/extracellular fluid and hence with Galvanic connection to the dura), higher currents are needed to reach electric fields high enough for stimulation at the same depth of the spinal cord as compared to the situation where the contact is in direct contact with the dura.

The localization of the observed functional activations in the brain as well as the effectiveness of OS-ESCS could be explained by considering the distribution of different tracts in the cross-section of the spinal cord, and also by taking into account the complex nature of the communication within the CNS. Specifically, the structures arranged in distance from the center of the stimulation are the bilateral dorsal columns, which carry sensory information to the brain, corticospinal tract in the dorsal section which carry descending information from the motor cortex^34^, the dorsal horn (lamina 1–6), the lateral columns, the intermediate grey matter (lamina 7), the area around central canal (lamina 10), the ventral horns (lamina 8–9), the ventral columns, and finally, located laterally to the electrode, the dorsal roots which contain a subgroup of neural fibers continuing into the dorsal columns and carrying sensory information, and a subgroup ascending to the thalamus carrying nociceptive information. Therefore, ascending and descending pathways relaying information to/from different regions in the brain might explain the activated areas in our group of animals. Although a multitude of pathways exist between the spinal cord, thalamus and motor cortex, most likely candidate sites for ESCS-related initiation of action potentials are the dorsal columns and the dorsal roots^35–37^. This is because of their shorter physical distance to the dorsally-placed epidural electrode and the presence of large diameter myelinated axons. Nonetheless, since electrical stimulation in the course of an axon potential elicits both centrifugal and centripetal signals^38^, the size and high myelination of corticospinal tract make it also one of the potential pathways carrying ESCS signals to the brain, and, specifically, to the motor cortex.

On the other hand, the corticospinal tract is a complex system targeting different regions of the spinal cord and its descending projections are known to play a major role in motion^39^. However, this system is also involved in a selective and complex modulation process of sensory feedback^39^ that we can hypothesize triggering motor commands at network level and, thus, leading to activation in the motor cortex, even without a direct stimulation of this region^40^ but via sensory or sensory-triggered responses in the motor cortex. Thus, it is plausible that motor cortex activation is part of a complex circuit that not only controls (and is activated by) motion stimuli but also receives sensory inputs from the periphery^40^.

The ability to selectively activate various pathways of the spinal cord may offer major advantages for multiple clinical applications. For pain management, it has been shown that targeting the dorsal columns and avoiding stimulating the dorsal roots is the best strategy^41^. It was suggested to place the contacts right above the midline of the spinal cord, which would maximize the distance to the dorsal roots^35,37,42^. Further optimization has been found with tripolar electrodes with two anodic guards placed laterally with the cathode in the middle, which allowed focusing the stimulation into the dorsal columns^43,44^. Such objective can be achieved with the OS-ESCS strategy suggested in this study. Although avoiding stimulating the dorsal roots for pain management is desired, dorsal root stimulation is on the other hand beneficial for restoring motor function after a spinal cord injury^7,8,45^. The trajectory of the dorsal roots varies from segment to segment and also from the lateral to medial sides, so that near the spinal cord dorsal roots gradually change the angle^33^. Therefore, the myelinated axons traversing rostrocaudally unavoidably change their orientation relatively to the stimulating electric field. This complex variation in the dorsal roots spatial orientation makes it challenging to identify an optimal orientation of electrical field gradient, which likely should be designed in a segment-specific manner^46^.

An interesting observation of our study is about the shape of the signal time courses, which in some dataset manifested not only an increase of amplitude, but also a delayed and pronounced undershoot (Supplementary Figure 7). The precise nature of this negative peak is not immediate to define. However, to qualitative characterize these negative peaks following the positive ones, we analyzed a representative dataset with the deconvolution method^47^ that allows to compute functional activation maps in a way that is independent from the shape of the hemodynamic response function (HRF). This method computes one map for each repetition time after the stimulus presentation, by applying the deconvolution-based general linear model (GLM) to the signal time-course with 12 “stick” predictors, 6 overlapping with the stimulus presentation and 6 overlapping with the first resting period (18 s). In line with the signal time courses, this representative case presents a pattern of positive responses after 3TR (9 s) and a consecutive pattern of negative responses after 8TR (24 s) (Supplementary Figure 7). Such observations warrant future investigations that will elucidate the parameters and conditions which elicit the negative responses, with the ultimate goal of establishing the HRF in general settings.

The current study also presents several limitations. First and foremost, the sample size was relatively small. To mitigate these limitations, we reported the statistical results at a strict statistical threshold (p < 0.05 FWE corrected for the main effects, p < 0.001 uncorrected at peak level but FDR corrected at cluster level for the one-vs-all analyses). However, the limited number of studies used for stimulating the S1 and L2 spinal segments, along with the different types of electrode used for the two sub-groups (tungsten and stainless steel wires, respectively), likely precluded the detection of group differences in brain patterns during OS-ESC of the two sites. Therefore, conclusive characterization of the distinct brain responses to S1 and L2 OS-ESCS would require dedicated studies with larger cohorts and consistent electrodes.

Another limitation of the study lies in the use of anesthesia during the fMRI session, namely urethane in our case. Although urethane has negligible effects on fMRI results and most closely resembles the awake condition^48^, it can induce switching between states of sleep during long experimental sessions like ours, thus potentially confounding the results. To exclude such a possibility, we calculated the group mean effects after performing an ICA analysis of each animal and angle. The goal of this approach was to select the ICA map having the corresponding time course best fitting the experimental paradigm, thus excluding other signal fluctuations which may have resulted from various factors of non-interest such as anesthesia. As reported in the Supplementary Figure 6, the activation patterns revealed in these analyses encompassed similar areas as those observed with the GLM approach, thus confirming that anesthesia did not significantly affect the overall results.

The relatively simple 3-channel design, and the relatively large size of the wires as compared to the dimension of the rat spinal cord, pose additional limitations to the current study. Future efforts will explore the feasibility of OS-ESCS with more advanced high-density electrodes custom-designed for specific applications.

Also, whereas fMRI is an invaluable tool to measure integrative responses of the CNS to ESCS, it only provides indirect measures of neural activity. Future comprehensive assessments could thus benefit from direct measurements of neuronal recruitment by electrophysiological recordings to be combined with fMRI or with functional ultrasound (fUS) outcomes. In our previous studies with electrophysiological recording during epidural stimulation we demonstrated that variable patterns of muscle activations depended on the position of the electrodes relatively to the dorsal roots^33^. Similarly, the neural-vascular coupling assessed with fUS was found to depend on the specific anatomical organization of the spinal cord^49^. Such observations substantiate the conceptual framework of OS-ESCS. fUS was also found to detect spinal cord hemodynamic changes during epidural stimuli which did not elicit peripheral motor responses^50^, demonstrating that electromyography may be inadequate to reveal the involvement of the spinal cord circuitry and warranting the use of integrative CNS approaches such as brain fMRI utilized here. fMRI of the spinal cord itself also warrants further consideration; however substantial electrode-induced artefacts need to be minimized to achieve robust responses. Such goal could be accomplished with zero echo time MRI sequences as used in our previous OS-DBS studies^16,18,51^.

In future studies, OS-ESCS paradigms could be combined with other advanced methods such as burst stimulation or high frequency stimulation, and their impact on behavioral outcomes for pain management or motor function could be evaluated. It should also be mentioned that clinical ESCS electrodes and human anatomy are different compared to those of rats. Hence, it is likely that the electric fields in patients would not behave exactly as in our study. Further work is needed using large animals and simulations to ensure that OS-ESCS principles can be used as intended in patients.

To conclude, this is the first brain fMRI study conducted during OS-ESCS. A distinct advantage of the orientation selective stimulation approach involves the added flexibility of targeting not just the stimulation site per se, but also specific axonal fiber orientations passing by or in close vicinity to the stimulation site with the ultimate goal of achieving optimal and selective activation of a circuitry of interest. This degree of freedom can be beneficial for maximizing the activation of connected downstream brain areas, possibly even mitigating the effects of minor electrode placement inaccuracies which are unavoidable in clinical practice. The current work also demonstrates that whole-brain fMRI can effectively monitor integrative CNS responses during OS-ESCS, and can thus help define optimal settings including the stimulation angle itself. The whole conceptual framework is easily translatable to clinical settings. Indeed, fMRI has been already used with SCS at 1.5 T^26^, and OS-ESCS paradigms can be implemented with available commercial electrodes that utilize independently driven channels such as those used in the Illumina3D system from Boston Scientific, similarly to what we demonstrated in the DBS arena^19^.

## Methods

### Surgical Procedures and Electrode Implantations

All surgical and experimental procedures were approved by the Institutional Animal Care and Use Committee of the University of Minnesota. All procedures were carried out in accordance with the relevant guidelines. The study was carried out in compliance with the ARRIVE guidelines. Male Sprague–Dawley rats (Envigo) aged 3–6 months, approximately 300 g were used. A group of n = 6 rats underwent monopolar ESCS for studying the frequency dependence of brain activations during ESCS; n = 12 rats underwent OS-ESCS with 3-channel electrodes. Data from one animal of the OS-ESCS group were discarded due to technical problems during the acquisition, and were not included in the analyses, thus leaving a total of n = 6 rats for monopolar ESCS, n = 11 for OS-ESCS.

For the 6 rats undergoing monopolar ESCS and for 5 out of 11 rats undergoing OS-ESCS, the electrodes were constructed of Teflon coated stainless steel wire (diameter 100 µm). Contacts were created by removing 1 mm of the coating from one side of the wire under a microscope using a scalpel and a scale with 100 µm accuracy. For OS-ESCS, the contacts were arranged in a triangle with two rostral and one caudal contact. Distance between the two rostral contacts were ~ 1 mm and between the rostral and caudal contacts ~ 2 mm. For the other 6 out of 11 rats undergoing OS-ESCS, the electrodes were made as above but by using polyimide coated tungsten wires (diameter 127 µm). The contacts were created by carefully removing 1 mm of insulation around the wire using a scalpel.

After the induction of isoflurane anesthesia (5% induction, 2.0–3.5% maintenance; carrier gas O_2_/N_2_O 30/70%) in prone position for the surgery, lidocaine (2%) was liberally injected intracutaneously for analgesia. Next, vertebral levels were first identified with reference to superficial markers of bilateral iliac crests (cristae iliacae) and lower costal margins. After shaving the skin of the back, posterior midline skin incision was made with target vertebral level at the center and extended no less than one level both superiorly and inferiorly. Para-vertebral back muscles were dissected exposing spinous processes and laminae of three continuous vertebral levels. For the monopolar ESCS studies (n = 6) and for the OS-ESCS studies using stainless steel electrodes (n = 5), the target was set to L2 vertebral level (corresponding to S1 spinal segment). Laminectomy surgery was performed for each case within this group. Lamina of L2 vertebral level was removed. Electrodes were placed at L2 vertebral level rostrally, closely in contact with the dura. Free soft tissue moistened with saline was put on top of the electrodes to ensure stable contact of the electrodes to dura. Spinous process of the lower level, L3, was removed since it would serve as a barrier in the route of tail wire connected to the electrodes. Terminal end of the wire was looped and sutured to the skin for fixation and prevention of being pulled out during subsequent procedures. The skin was closed loosely. For the OS-ESCS studies using tungsten electrodes (n = 6), the target was set to T13 vertebral level (corresponding to L2 spinal segment), chosen in attempt to further reduce movement artefacts during fMRI while still targeting the lumbosacral circuitry. Lamina-preservation surgery was used for this group. Enlarged windowing was made at both superior and inferior inter-laminar spaces around the target level lamina (T13). Spinous process of L1 was removed for the convenience of wire routing. Epidural space under T13 lamina was cleared. The electrodes were implanted rostrally beneath T13 lamina, with the tip reaching the rostral level of T13 lamina. Free soft tissue moistened with saline was used to cover the exposing part of the electrodes. Subsequent procedures were the same as for the laminectomy surgery, the terminal end of wire was fixed and the skin was closed. For all animals, a separate Ag/AgCl ground electrode was implanted subcutaneously through another incision approximately 1–2 cm away from stimulation electrodes. Ag/AgCl was chosen for the ground electrode because of its non-polarizable nature.

After surgery, anesthesia was switched to urethane (4 consecutive intraperitoneal injections with the dose of 1.25–1.50 g/kg of body weight, 15 min apart) while gradually decreasing the isoflurane level and discontinuing it at the last urethane injection. During surgery and in the MR scanner, the temperature and the respiration of each rat were monitored by optical rectal temperature probe and pressure respiration sensor. The temperature was maintained at 37 °C by a heating pad during surgery, and with heated water circulation and heated air during the MRI scan. After MRI, high-resolution CT scans were acquired from 4 out of the 6 rats implanted with tungsten 3-channel electrodes to image the location of the electrode. Images were acquired with 4 × 4 binning, 0.5 mm filter/full rotation 360 projections, 80 kV, 500 µA, 180 ms acquisition time, for 87 µm isotropic resolution. 3D reconstructions of CT images were performed with Feldkamp using down-sampling 2, leading to 175 µm isotropic nominal resolution (Supplementary Figure 1).

### MRI data acquisition and stimulation paradigm

All MRI scans were conducted with a 9.4 T 31-cm horizontal-bore magnet equipped with Agilent DirecDRIVE console (Palo Alto, CA, USA) using a quadrature RF coil designed for full rat brain coverage. Before fMRI, anatomical images were acquired using a Fast Spin-Echo (FSE) sequence with the following parameters: repetition time (TR) = 3 s, effective echo time = 48 ms, number of echoes = 8, FOV = 32 × 32 mm^2^, 15 slices with 1 mm thickness. fMRI was conducted using the spin-echo echo planar imaging (SE-EPI) MRI pulse sequence with TR = 1.5 s, TE = 37 ms, two shots, FOV = 32 × 32 mm^2^, 0.5 × 0.5 mm^2^ in plane resolution, 15 slices with 1 mm thickness and 98 repetitions (effective TR = 3 s). Stimulation paradigm consisted of 3 blocks of 60 s of rest and 18 s of stimulation using cathodic 500-µs symmetric biphasic square pulses. First, using a single contact/wire, monopolar stimulation frequencies of 5, 20, 40, 80, 160, 320 and 640 Hz were tested. Next, OS-ESCS was applied in a different set of animals using a stimulation frequency of 40 Hz based on the initial evaluation of maximal fMRI response in the thalamus. The current amplitude of stimulation was chosen in a subject specific way, through preliminary fMRI scans by applying monopolar stimulation at 40 Hz frequency and choosing the current level giving a non-artefactual blood oxygen level dependent (BOLD) responses in the brain.

### Simulation of OS-ESCS in rat spinal cord

The electric fields generated by the OS-ESCS paradigms were simulated with a finite element methods model. An anatomically realistic model of the spinal cord near the target area of stimulation was constructed based on Watson atlas^52^ using SolidWorks 2018 (Dassault Systèmes, Waltham, MA), after which the model was transferred to COMSOL 5.4 (COMSOL, Stockholm, Sweden). Tissue surrounding the spinal cord was modelled as concentric cylinders (height = 20 mm, diameter = 10 mm) representing bone (thickness d = 1 mm, conductivity σ = 0.02 S/m), muscle (d = 1.5 mm, σ = 0.25 S/m), epidural fat (d = 0.8 mm, σ = 0.04 S/m), dura (d = 0.1 mm, σ = 0.6 S/m) and CSF (d = 0.1 mm, σ = 1.7 S/m)^53^. The width of the spinal cord varied from 3 to 4 mm between the rostral and caudal ends of the simulated volume. The conductivity of the grey matter was modelled as isotropic (σ = 0.23 S/m), while the conductivity of the white matter was modelled as anisotropic (σ_parallel_ = 0.6 S/m, σ_perpendicular_ = 0.083 S/m). To model the effect of the surgery, a sector of the surrounding tissue above the dura was removed off the top of the anterior spinal cord. The electrode contacts were modelled as 3 cylinders according to the dimensions described above and placed above the dura so that the curved surfaces of the cylinders conducted while the flat ends were isolated using the Neumann boundary condition. Ground was set to the bottom of the model and remaining boundaries were isolated. The nominal current amplitude was set to 1 mA and current was delivered through the contacts according to Eq. (1) (see below) using stimulation angles 0°, 30°, 60°, 90° and 180°. The detailed description of OS using a 3-channel electrode is given in Lehto et al.^17^. Briefly, in our experimental protocol the orientation of electric field was set to varying angles between 0° (dipole cathodic lobe towards the head) and 330° with steps of 30° using a multichannel configuration. To orient electric field dipole, the current amplitudes of 3 electrodes were selected according to the equations below (Eq. 1):

1 $$\begin{aligned} I_{1} & = I_{0} cos\left( {\emptyset + 120^\circ } \right) \\ I_{2} & = I_{0} cos\left( \emptyset \right) \\ I_{3} & = I_{0} cos\left( {\emptyset - 120^\circ } \right), \\ \end{aligned}$$

where $I_{1,2,3}$ are the current amplitudes contacts 1 to 3, $I_{0}$ is the stimulation current amplitude and $\emptyset$ governs the stimulation angle.

The angles of stimulation were set such that 0°/180° corresponds to the rostral-caudal direction while − 90°/90° corresponds to the left–right direction, respectively.

### fMRI data processing and statistical analysis

Anatomical and functional SE-EPI data were analyzed with the SPM8 toolbox (http://www.fil.ion.ucl.ac.uk/spm/) running on MATLAB 7.6 (The MathWorks, Inc., Natick, MA, http://www.mathworks.com). For each subject, the anatomical data was normalized to an animal template outside the fMRI group based on FSE images. Functional data were first corrected for slice timing and for motion artifacts, and then coregistered to the corresponding anatomical data and finally normalized to the animal template using the transformation of the anatomical data. Mean framewise displacement was also calculated for each rat and each functional run as defined in Power et al.^54^, and 0.15 mm was set as threshold for exclusion. Finally, the functional data was smoothed with a full width half maximum (FWHM) Gaussian kernel (size 1 functional voxel). The single-subject analyses, performed for both frequency-related and OS-ESCS stimulation, were computed using a GLM that consisted of a block design model convolved with a first-order gamma HRF of 15 s length^55^. During GLM fitting, functional data were high-pass filtered in the temporal domain using a cut-off of 1000 s. A correction for serial correlation was applied using a first-order auto-regressive model applied to the GLM residuals.

Beta maps and contrast t-maps maps were computed. The beta maps of the single-subject analysis were used for the two separate group-based statistical analyses, namely monopolar ESCS (n = 6, frequency dependence) and OS-ESCS (n = 11, angle dependence). A one-way within-subject ANOVA model was applied with the frequency or stimulation angle defined as factor (7 levels for monopolar ESCS, 12 levels for OS-ESCS). The stimulation currents, and in the case of OS-ESCS also the two experimental setups (namely, setup 1: S1 spinal segment stimulation with stainless steel electrodes, and setup 2: L2 spinal segment stimulation with tungsten electrodes), were added as covariates. Stimulation currents were adjusted for each angle to reflect the actual current flowing across contacts according to Eq. (1). Maps of main effects for both single subjects and group-based analyses were finally computed after applying a statistical threshold of p < 0.05 FWE for the group-based OS-ESCS and for the monopolar group-based ESCS frequency-related experiments, and a statistical threshold of p < 0.001 uncorrected for the single-subject OS-ESCS maps. ANOVA analyses were repeated also in absence of co-variates to assess the impact of stimulation currents and experimental setups on the main group effects.

For the OS-ESCS group, we additionally performed a “one-vs-all” contrast analysis to determine brain areas where activations obtained at a certain angle were significantly higher as compared with all the other directions. This was achieved by calculating the differential contrast “one vs all” of beta-maps at each angle vs all other angles. The contrast statistical t-map was thresholded at p < 0.001, and significant clusters were identified whether they survived the threshold of p < 0.05 FDR corrected.

Average beta-values were additionally computed in anatomical defined regions of interest (ROIs), namely the thalamus, the motor cortex and the primary somatosensory cortex, and were statistically analyzed per ROI using a linear mixed model with fixed effects for angle or frequency, stimulation currents and experimental setups (in the angle model only) as covariates and random effects for rats (random intercept only). Each angle was compared to 0°, and each frequency was compared to 5 Hz. Holm’s correction was applied for correcting the multiple pairwise comparisons between angles or frequencies.

BOLD signals in each ROI were also computed by dividing the signal time course for its baseline, calculated as mean of the last 5 volumes during the rest periods after each stimulus repetition. The resulting signals, one for each stimulation angle, were averaged across rats and plotted as mean ± standard deviation.

For evaluating possible biases induced by anesthesia on the functional time-series, we analyzed the fMRI data after conducting ICA, as described previously^18^. Briefly, ICA was performed using MELODIC tool of FSL (https://fsl.fmrib.ox.ac.uk/fsl) to extract 16 ICA maps for each animal and each angle. We visually inspected all the ICA components to select the maps having the corresponding signal time course best fitting the stimulation paradigm. In this way for each rat we collected 12 ICA maps (one for each experimental condition/angle) that were considered in the one within ANOVA model with experimental setups and stimulation currents as covariates of no-interest.

Lastly, in order to assess differences in activation patterns induced by the two experimental setups, we computed a flexible factorial design with the angle as one-within factor and the two groups as one-between factor (Group 1: 5 animals undergoing setup 1, group 2: 6 animals undergoing setup 2). The same one-within/one-between ANOVA analysis was performed at regional values in the thalamus, motor cortex and primary somatosensory cortex on the mean beta values after regressing out the stimulation currents.

## Supplementary Information

Supplementary Figures.

## Data Availability

The datasets generated during and/or analyzed during the current study are available from the corresponding author on reasonable request.
