# Supplementary material for: Brain fMRI during orientation selective epidural spinal cord stimulation
Source: Sci Rep. 2021 Mar 9;11:5504. doi: 10.1038/s41598-021-84873-8 (PMC7943775; doi:10.1038/s41598-021-84873-8)
Supplement: Supplementary file 1 — Supplementary Figures. [file 41598_2021_84873_MOESM1_ESM.docx]

**Supplementary Material**

**Brain fMRI during Orientation Selective Epidural Spinal Cord Stimulation**

Antonietta Canna^1,2^, Lauri J Lehto^1^, Lin Wu^1^, Sheng Sang^1^, Hanne Laakso^1,3^, Jun Ma^4^, Pavel Filip^1,5^, Yuan Zhang^6^, Olli Gröhn^3^, Fabrizio Esposito^2^, Clark C Chen^4^, Igor Lavrov^7,8^, Shalom Michaeli^1^, Silvia Mangia^1*^

^1^Center for Magnetic Resonance Research (CMRR), Department of Radiology, University of Minnesota, Minneapolis, MN, USA

^2^Department of Advanced Medical and Surgical Sciences, University of Campania “Luigi Vanvitelli”, Napoli, Italy

^3^A. I. Virtanen Institute for Molecular Sciences, University of Eastern Finland, Kuopio, Finland

^4^Department of Neurosurgery, University of Minnesota, Minneapolis, MN, USA

^5^First Department of Neurology, Faculty of Medicine, Masaryk University and University Hospital of St. Anne, Brno, Czech Republic

^6^Division of Biostatistics, School of Public Health, University of Minnesota, Minneapolis, MN, USA

^7^Department of Physiology and Biomedical Engineering, Mayo Clinic, Rochester, MN, USA

^8^Department of Neurology, Mayo Clinic, Rochester, MN, USA

*Corresponding author:

Silvia Mangia, PhD

University of Minnesota, Radiology Department

Center for MR Research 2021 6th St. SE

Minneapolis, MN 55455

Phone: (612)-626-2001

E-mail: mangia@umn.edu

FAX: (612)-626-2004

**
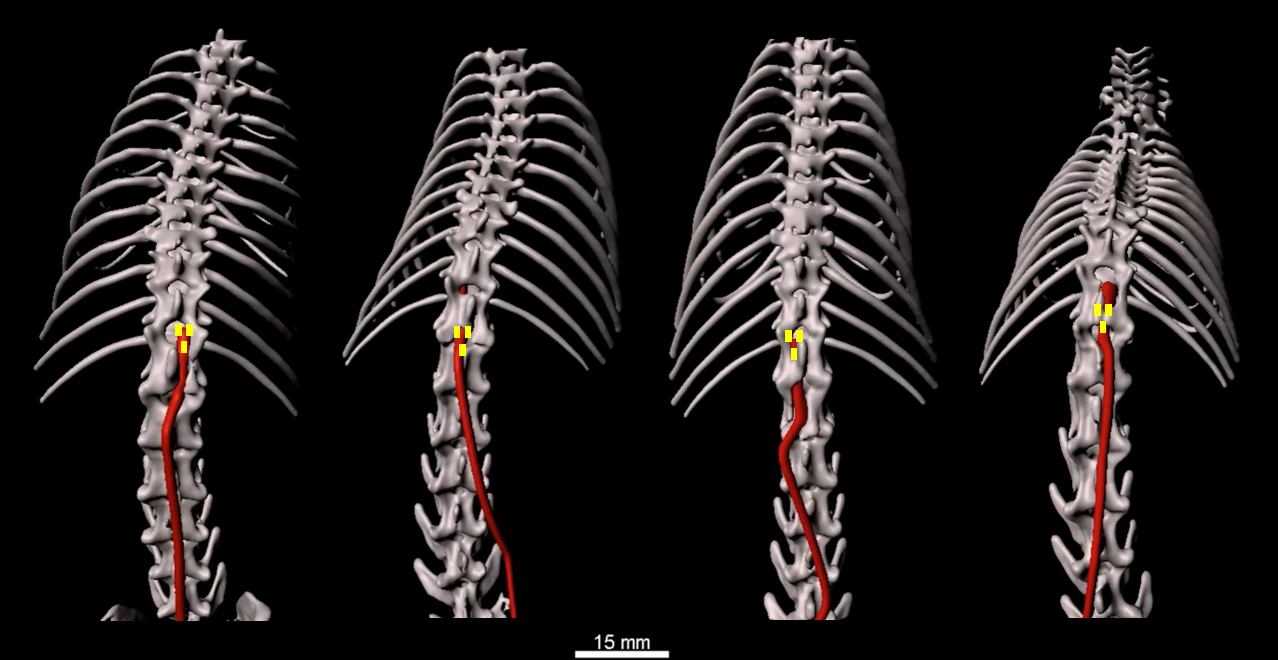
Supplementary Figure 1.** 3D reconstructions of high-resolution CT scans acquired at the end of the study for documenting the location of the implanted electrode**.** The three yellow squares represent the leads of the electrodes. For the animals illustrated here, the implantation target was the L2 spinal segment (T13 vertebral level).

**
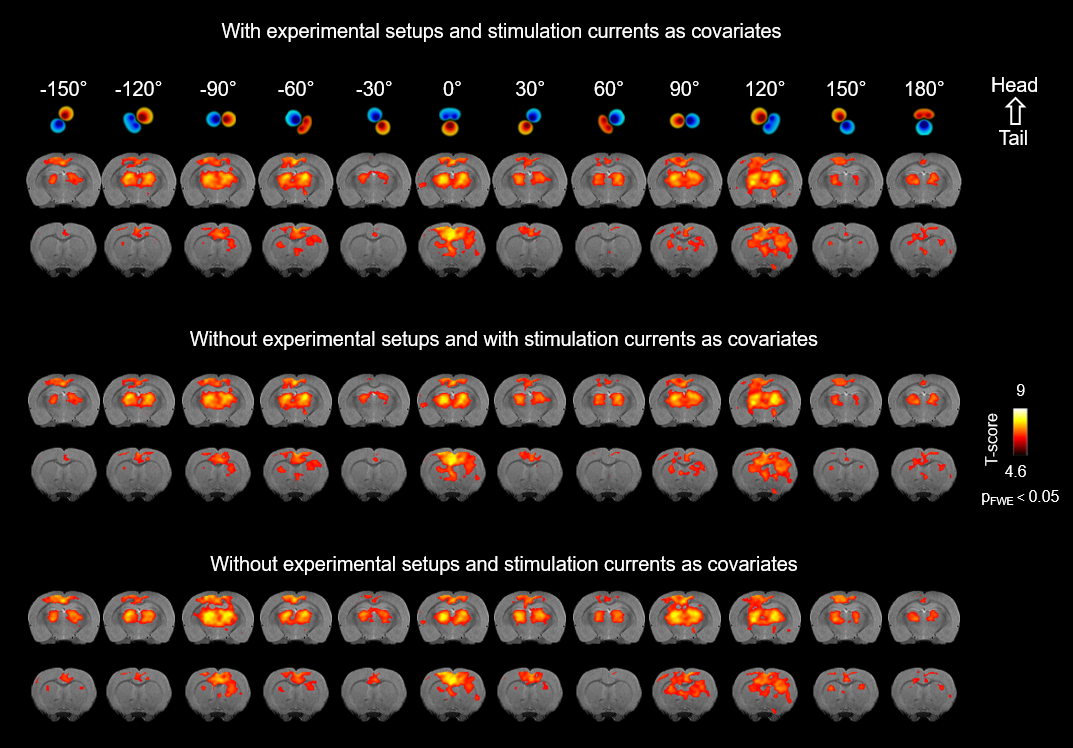
**

**Supplementary Figure 2:** Group level maps of the main effects (t-maps) obtained with the one-between ANOVA analysis with experimental setups (namely, setup 1: S1 spinal segment stimulation with stainless steel electrodes; setup 2: L2 spinal segment stimulation with tungsten electrodes) and stimulation currents as covariates (top), without experimental setups and with stimulation currents as covariates (middle), and without experimental setups and stimulation currents as covariates (bottom). High similarity of the resulting maps can be appreciated. Brain images are displayed in neurological convention (left side of the image corresponds to the left side of the brain).


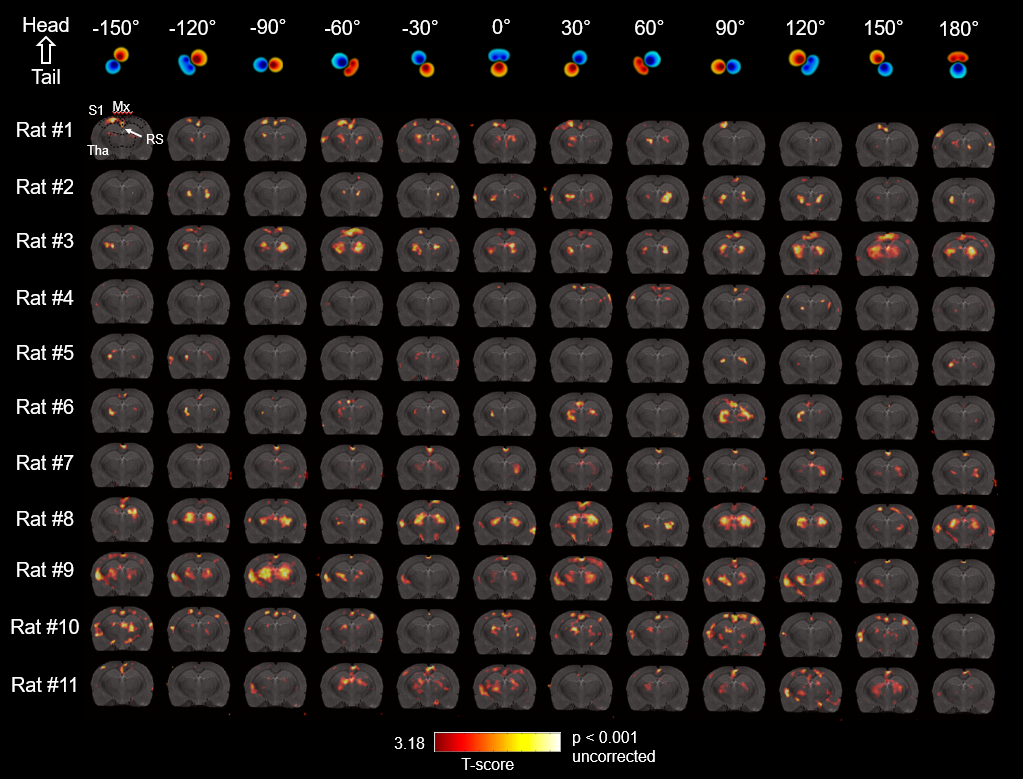


**Supplementary Figure 3.** Individual maps of the main effects (t-maps) in a brain slice encompassing the primary somatosensory cortex (S1), motor cortex (Mx, including primary and secondary motor cortices), thalamus (Tha), and retrosplenial (RS). Contrast t-maps were obtained by the first level GLM analysis in response to OS-ESCS. Brain images are displayed in neurological convention (left side of the image corresponds to the left side of the brain).


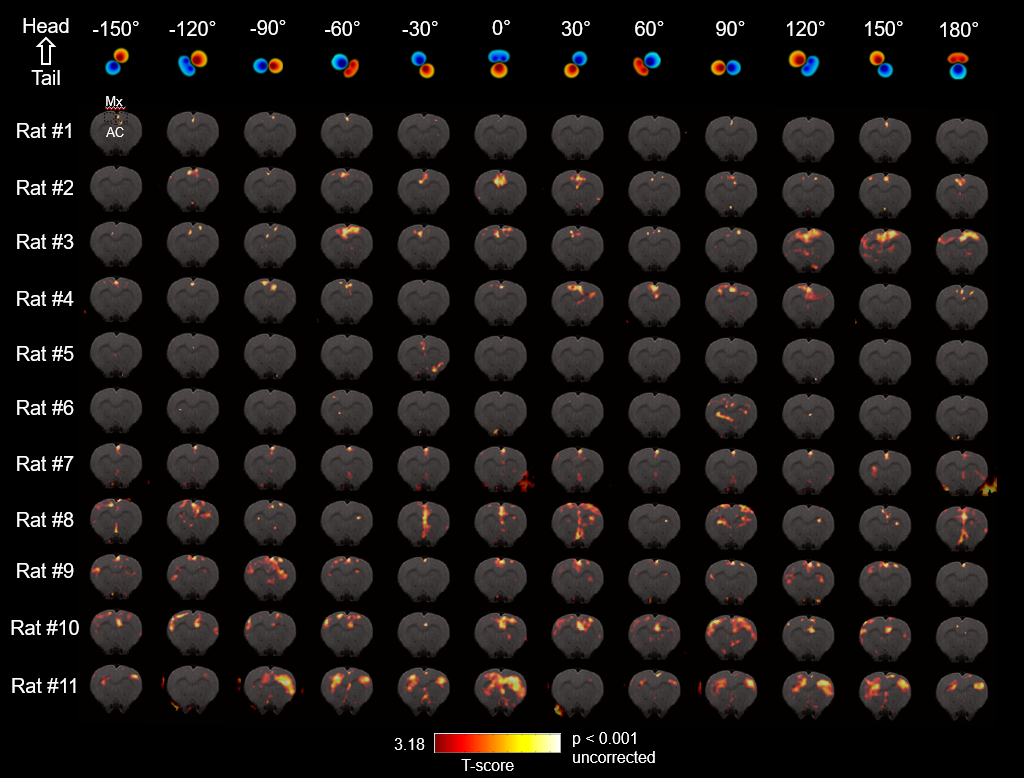


**Supplementary Figure 4.** Individual maps of the main effects (t-maps) in a brain slice encompassing the primary and secondary motor cortex (Mx) and anterior cingulate cortex (AC). Contrast t-maps were obtained by the first level GLM analysis in response to OS-ESCS. Brain images are displayed in neurological convention (left side of the image corresponds to the left side of the brain).

**
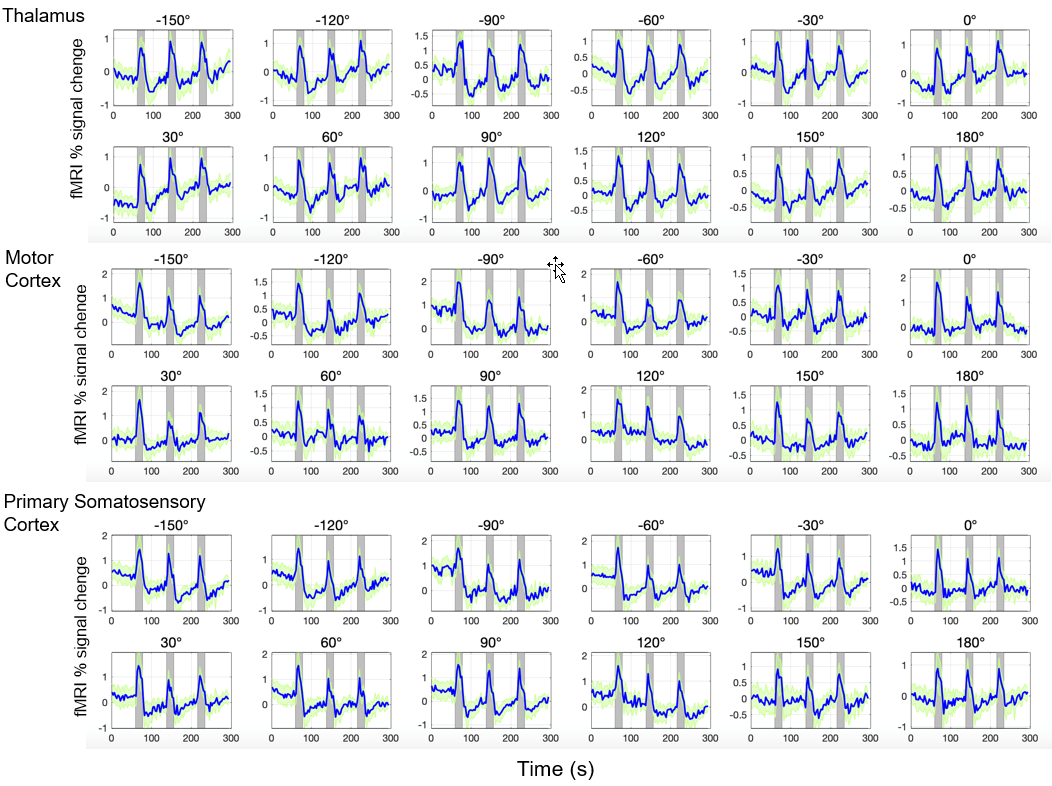
**

**Supplementary Figure 5.** fMRI signal changes computed by dividing the signal time course by its baseline, the latter calculated as mean of the last five volumes of each stimulus repetition. The resulting signals, one for each stimulation angle, were averaged across rats and plotted as mean ± standard deviation. Blue line indicates mean while green shading indicates the standard deviation. Gray boxes indicate the stimulation paradigm.

**
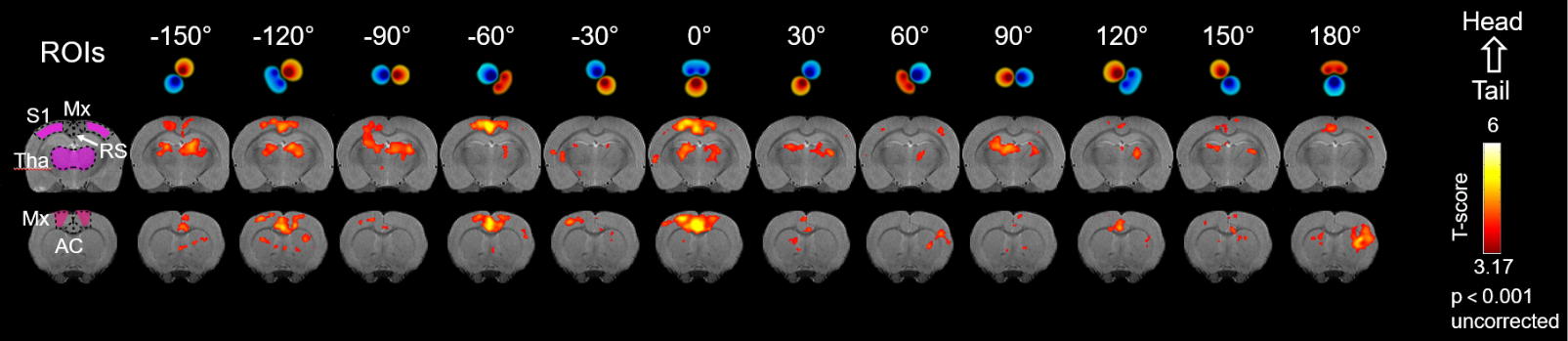
**

**Supplementary Figure 6:** Results of the one-between ANOVA analysis with experimental setups (namely, setup 1: S1 spinal segment stimulation with stainless steel electrodes; setup 2: L2 spinal segment stimulation with tungsten electrodes) and stimulation currents as covariates of no interest performed on the ICA maps in the group of 11 rats considered for the OS-ESCS experiment. Two different brain slices are shown, encompassing the S1, primary somatosensory cortex; Mx, motor cortex (including primary and secondary motor cortices); RS, retrosplenial; Tha, thalamus; AC, anterior cingulate cortex. Brain images are displayed in neurological convention (left side of the image corresponds to the left side of the brain).

**
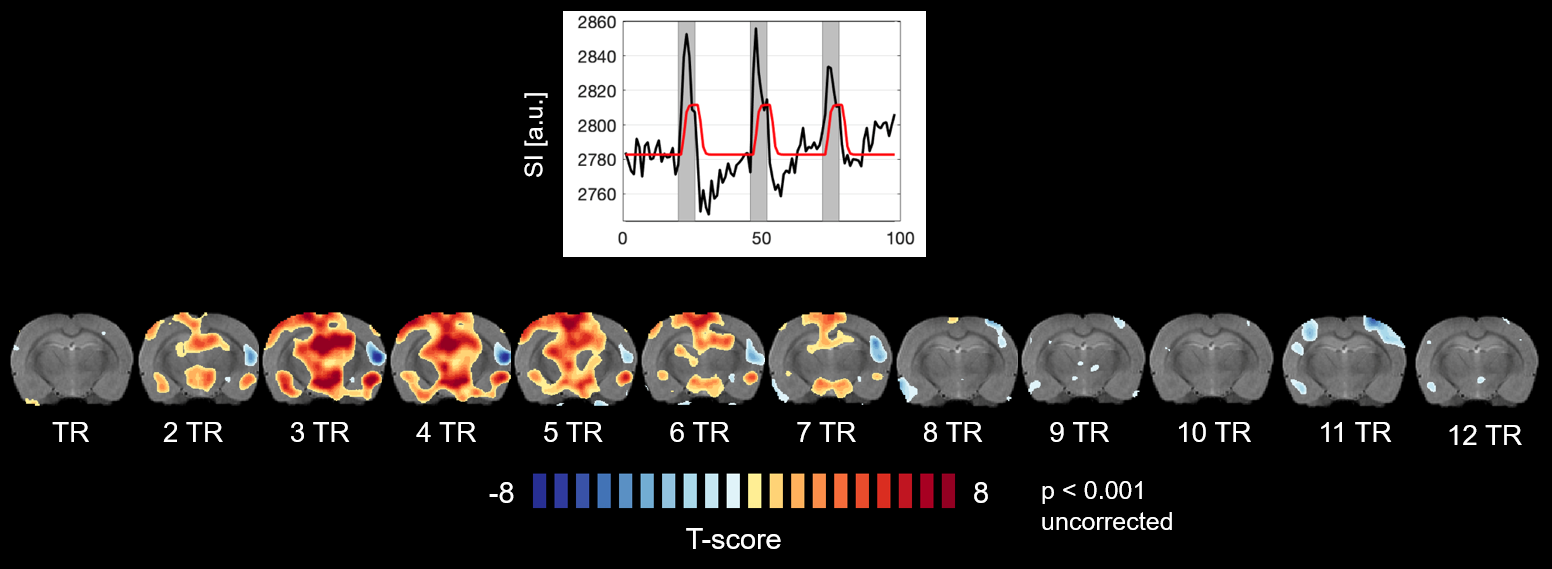
**

**Supplementary Figure 7**: First row indicates a representative signal time course (black line), and the predictor used in the SPM analysis (red line). The second row shows the functional maps obtained by processing the dataset with the deconvolution approach and 12 sticks predictors. Brain images are displayed in neurological convention (left side of the image corresponds to the left side of the brain).
